# Testing and Prediction of Shear Performance for Steel Fiber Reinforced Expanded-Shale Lightweight Concrete Beams without Web Reinforcements

**DOI:** 10.3390/ma12101594

**Published:** 2019-05-15

**Authors:** Xiaoke Li, Changyong Li, Minglei Zhao, Hui Yang, Siyi Zhou

**Affiliations:** 1 International Joint Research Lab for Eco-building Materials and Engineering of Henan, North China University of Water Resources and Electric Power, Huanyuan Campus, No. 36 Beihuan Road, Zhengzhou 450045, China; zxyanzi@ncwu.edu.cn (H.Y.); syzhou@stu.ncwu.edu.cn (S.Z.); 2 Henan Provincial Collaborative Innovation Center for Water Resources High-efficient Utilization and Support Engineering, No. 136 Jinshui East Road, Zhengzhou 450046, China; 3 School of Engineering, RMIT University, Melbourne, VIC 3003, Australia; coffeyha@aliyun.com

**Keywords:** steel fiber reinforced expanded-shale lightweight concrete (SFRELC), reinforced SFRELC beam, shear cracking resistance, shear capacity, shear-span to depth ratio, volume fraction of steel fiber, longitudinal reinforcement ratio, SFRELC strength, prediction formulas

## Abstract

In this paper, for a wide application of high-performance steel fiber reinforced expanded-shale lightweight concrete (SFRELC) in structures, the shear behavior of reinforced SFRELC beams without web reinforcements was experimentally investigated under a four-point bending test. Twenty-six beams were fabricated considering the influencing parameters of SFRELC strength, shear-span to depth ratio, longitudinal reinforcement ratio and the volume fraction of the steel fiber. The statistical analyses based on the foundational design principles and the experimental results are made based on the shear cracking resistance, the shear crack distribution and width, the mid-span deflection, the patterns of shear failure, and the shear capacity of the specimens. This confirms the effective strengthening of steel fibers on the shear performance of reinforced SFRELC beams without web reinforcements. Based on the modifications to the formulas of reinforced conventional concrete, lightweight-aggregate concrete or steel fiber reinforced concrete (SFRC) beams, and the validation against the experimental findings, formulas are proposed for the prediction of shear cracking resistance and shear capacity of reinforced SFRELC beams without web reinforcements. Finally, formulas are discussed for the reliable design of the shear capacity of reinforced SFRELC beams without web reinforcements.

## 1. Introduction

With the development of modern concrete technology, lightweight concrete reinforced by steel fibers or synthetic fibers has become a popular issue in the research and application of civil engineering [1,2,3,4]. Due to its reliable mechanical properties [5,6,7,8,9], steady volume [10], enhanced durability [8,11], and rational bond performance to rebars [12], steel fiber reinforced expanded-shale lightweight concrete (SFRELC) can be applied as a structural material. One of these attempts is applying SFRELC to the prefabricated structural members in tensile zone under loadings. It was verified that the cracking resistance and loading capacity of beams under static and fatigue loads can be strengthened [13,14,15], and the punching shear ductility and resistance of superposed slabs can be significantly improved [16]. To increase the efficiency in prefabrication, transportation, and installation of precast members, reinforced SFRELC beams were developed, and their flexural behavior was experimentally investigated [17]. Results showed that the cracking moment, the flexural stiffness, the flexural capacity, and the flexural ductility of reinforced SFRELC beams were effectively improved due to the addition of steel fibers, and the crack growth was restrained with decreased space and width.

In view of the shear performance, the key issue concentrates on the shear capacity related to the crack distribution and the failure ductility. This should be evaluated based on the design principle of reinforced concrete structures and the reference of the standards for reinforced lightweight-aggregate concrete beams [18,19,20], reinforced conventional concrete beams [21,22], and steel fiber reinforced concrete (SFRC) beams [23,24]. Theoretically, when a main shear crack forms at a shear-span, the transfer of shear force in reinforced conventional concrete beams without web reinforcements occurs by a combination of the following principal mechanisms [25,26,27,28,29,30,31]:

(1) Shear resistance of the uncracked concrete in a compression zone. This part relies on the depth of the compression zone to provide bearing for block of uncracked concrete. With the increase of shear-span to depth ratio, the load bearing pattern transfers from inclined compression, to shear compression to diagonal tension; further, no depth of uncracked block exists on top zone with the change of failure modes mentioned above. Therefore, this part of shear resistance decreases with the increasing shear-span to depth ratio.

(2) The interlocking action of aggregates along the cracked concrete surfaces on the sides of the crack. This part depends on the roughness of the concrete surfaces affected by many factors, such as the types of coarse aggregate, the strength of the cement mortar, and the inclined angle of the crack.

(3) The dowel action of the longitudinal reinforcement. This provides the linkage of the cracked concrete and transfers the tensile force of the longitudinal reinforcement along the depth and span to contribute to the shear resistance.

(4) The simultaneous occurrence of both arch action and beam action. The arch action results from a constant tensile force of longitudinal reinforcement acting along a varying internal lever arm, while the beam action results from a gradient in the tensile force of longitudinal reinforcement on a constant internal lever arm. The arch action contributes mainly to a short beam with a shear-span to depth ratio of 1 < *λ* ≤ 2.5, and the beam action is the governing characteristics of a slender beam with *λ* > 2.5.

Based on above mechanisms, the shear-span to depth ratio, the reinforcement ratio of longitudinal rebar, and the strength of concrete were normally selected as the main influencing factors to build semi-empirical formulas for computing the shear capacity, although great variation existed in the experimental results due to many influencing factors and complex mechanism. As different datasets were used to set up the forms and determine the values of coefficients in semi-empirical formulas, no unified formula was globally accepted [27,28,29,30,31,32]. In view of design reliability, different semi-empirical formulas representing the lower enveloping curve for test datasets have been adopted in different standards [19,20,21,22].

A similar research route was always adopted to study the shear performance of reinforced lightweight-aggregate concrete beams without web reinforcements. The factors mentioned above are always considered in experimental investigations to verify the adaptability of the same semi-expression of reinforced conventional concrete beams, while the coefficients of the semi-empirical formulas were modified by fitted analyses [31,32,33,34,35]. Compared to conventional concrete with a natural aggregate, the lightweight-aggregate concrete fails due to the rupture of the aggregates themselves rather than the interface between the aggregate and cement mortar. This leads to a smooth surface of cracks, and reduces the ability of interlocking action [33,34].

Meanwhile, the actions of steel fibers in conventional concrete mainly appeared in the studies of shear performances of reinforced SFRC beams without web reinforcement, and the semi-empirical formulas for shear capacity were proposed as two forms. One was based on the principle of steel fibers acting as a reinforcement, and the contribution of steel fibers to shear capacity was separated as one term added into the formulas of reinforced conventional concrete beams [24,26,36,37,38]. Another was based on the strengthening concrete with steel fibers, and the contribution of steel fibers to shear capacity was concluded in that of the concrete [23,26,28,39].

Relatively, few studies were carried out to study the shear performance of steel fiber reinforced lightweight-aggregate concrete beams. Swamy et al. [36] carried out tests on steel fibers used alone as shear reinforcement in full-sized lightweight-aggregate concrete I-beams with varying longitudinal tensile rebars and a shear span. Results showed that the fibers reduced the beam deformations substantially at all load levels, controlled the dowel and shear cracking, reduced the spalling in the cover, helped preserve the ductility and overall integrity of the structural member, and increased the ultimate shear strength by 60% to 200%. Flexural failure took place in beams with a lower ratio of longitudinal reinforcement and in beams with larger shear spans. Kang et al. [37] tested the steel fiber-reinforced lightweight-aggregate concrete beams without web reinforcements in conditions where the shear-span to depth ratio was 2, 3, and 4, and the volume fraction of the steel fiber was 0–0.75%. Results showed that the addition of a 0.75% volume fraction of steel fiber promoted a higher ductility and increased the shear capacity by 30%, and the shear-span to depth ratio adversely affected the shear capacity. Campione [38] proposed an analytical model that is able to determine the shear resistance of steel fiber-reinforced lightweight-aggregate concrete beams without web reinforcements. This model is based on the evaluation of the resistance contribution resulting from beam and arch actions. Results showed that the presence of steel fibers produces a further increase in shear capacity compared with reinforced lightweight concrete beams. Referring to the beam action, steel fibers ensured a higher shear capacity due to the increase in internal moment of the beam and the better bonding conditions of the longitudinal rebars. The arch effect is caused by the enhanced post-cracking resistance and the increase of the crack spacing along the main bars. Meanwhile, based on the validity analyses with the experimental data, prediction formulas for the shear capacity of steel fiber-reinforced lightweight-aggregate concrete beams without web reinforcement, compared with those of reinforced lightweight-aggregate concrete or conventional concrete beams, were proposed [36,37,38].

Based on the reviews above, as a fundamental content of structural design on reinforced SFRELC beams under shear load, the effectiveness of steel fibers on shear performance should be identified for the reliable design and safe application of SFRELC in concrete structures. For this purpose, experimental tests on 26 beams under a four-point bending test were carried out and the influences of different parameters, such as shear-span to depth ratio, longitudinal reinforcement ratio, volume fraction of steel fiber, and SFRELC strength were investigated. The shear cracking resistance, distribution and pattern of shear cracks, mid-span deflection, failure modes, and shear capacity of the beams are discussed in detail, combined with the principles of Materials Mechanics. Compared to the compressive and tensile strengths of the SFRELC specimens fabricated alongside the test beams, the actions of steel fibers in SFRELC on the shear performance of the reinforced SFRELC beams without web reinforcement are analyzed. Based on the modifications to the formulas of reinforced conventional concrete, lightweight-aggregate concrete or SFRC beams and the validation against the experimental findings, the predictive formulas for shear cracking resistance and shear capacity of reinforced SFRELC beams without web reinforcement are suggested. Finally, the design formulas of shear capacity with the reliability represented by the lower curve enveloping all test data are proposed.

## 2. Experimental Work

### 2.1. Preparation of SFRELC

The binder of SFRELC was grade 42.5 ordinary silicate cement; the compressive and flexural-tensile strengths were 50.8 MPa and 8.0 MPa at 28 days. The coarse aggregate was sintering expanded shale with maximum size of 20 mm, which was sieved in continuous gradation based on the maximum density principle [40]. The bulk and particle densities were 800 kg/m^3^ and 1274 kg/m^3^, the cylinder compressive strength was 5.0 MPa, the water absorption for 1 h was 6.1%. The lightweight sand was made of the byproduct of sintering expanded shale. The fineness modulus was 3.56 in continuous gradation with particle size of 1.6–5 mm, the bulk and particle densities were 946 kg/m^3^ and 1659 kg/m^3^, the water absorption for 1 h was 9.0%. Steel fiber was of milling type with a length *l*_f_ = 30 mm and an equivalent diameter *d*_f_ = 0.8 mm, the tensile strength was over 800 MPa. Other materials include the polycarboxylic acid super-plasticizer and tap-water. The mix proportion of SFRELC was calculated first in accordance with the absolute volume method specified in China cords [41,42], and adjusted reasonably based on previous studies [7,8,9,10]. The saturated dry surface condition of expanded shale and lightweight sand were adopted for the preparation of a fresh mixture of SFRELC, while their 1 h water absorptions were considered to compute the dosage of presoaking water.

### 2.2. Fabrication of Test Beams

Test beams were designed as rectangular cross-sections. To test the shear failure of reinforced SFRELC beams, the longitudinal tensile reinforcement was increased based on the design of reinforced conventional concrete beams [22,23]. Twenty-six test beams were designed as thirteen groups, and two of them were the same as a group. The section was 150 mm wide and 400 mm deep, and the length was 3200 mm. Two deformed rebars with diameter *d* = 22 mm, 25 mm, or 28 mm in each beam were used for the longitudinal reinforcement, and no web reinforcement was arranged. The concrete cover *c*_s_ = 25 mm, and the distance from the barycenter of longitudinal reinforcement to the bottom of cross-section *a*_s_ = *c*_s_ + *d*/2. The effective depth of the cross-section *h*_0_ = 400 − *a*_s_.

The main influencing factors considered in this study were shear-span to depth ratio (*λ*), SFRELC strength, longitudinal reinforcement ratio (*ρ*), and the volume fraction of steel fiber (*v*_f_). Table 1 presents their combination for the design of the test beams. Where a/b in the identifier represents the two beams in each group; *b* is the sectional width, *h*_0_ is the effective depth of cross-section, and *a* is the length of shear-span.

The single-horizontal-shaft forced mixer was used to mix the fresh mixture of SFRELC. The expanded shale and lightweight sand were firstly pre-soaked in the mixer for 1 h, and then the cement and half dosage of the mix water were added and mixed for 30 s. During the mixing, a super-plasticizer and another half dosage of mix water were added. After that, the steel fiber was sprinkled into the mixer and mixed for 3 min [7,8,9,10].

The mixture was cast into the steel form of the test beam for two times and compacted with vibrators fixed to the sides of the steel form. The screed top-surface was covered by plastic film for 48 h before being demoulded. After being cured with sprayed water for 7 days, the test beams were placed in a natural condition before testing. Standard specimens of six cubes with dimension of 150 mm and three prisms of 150 mm × 150 mm × 300 mm were fabricated and cured at the same condition as the test beams. They were tested for the cubic compressive strength (*f*_cu_), splitting tensile strength (*f*_ft_), and axial compressive strength (*f*_fc_) of the SFRELC used for the test beams. The tested values are presented in Table 1.

### 2.3. Test Method

**Figure 1:**
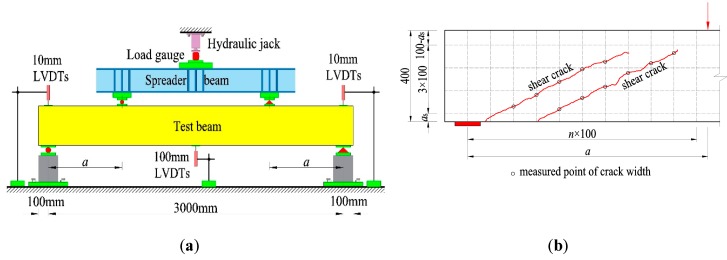
Test setup and measurement of shear crack width: (**a**) test setup; (**b**) measurement of shear crack width.

A four-point bending test was carried out. Two concentrated loads were exerted on the top-surface of the test beam by hydraulic jacks fixed on the loading frame, as presented in Figure 1a. The value of the load was measured by the loading sensor. The loads corresponding to shear-cracking resistance and failure were recorded, and the tested shear-cracking force *V*_cr_ and shear capacity *V*_cu_ are summarized in Table 1. In this study, the initial shear crack was determined as an inclined web crack appeared on the shear-span, or a shear-flexural crack at the bottom of shear-span turned to elongate to the load point. Three displacement sensors (LVDTs—linear variable differential transformer) were placed at the supports and mid-span section to measure the mid-span deflection of test beam. As presented in Figure 1b, the grid was drawn on the side surface of test beam. At the points where the shear crack intersected the vertical lines of the grid, the shear crack width was detected with a reading microscope by matching the crack with scale lines on the lens.

## 3. Discussion of Test Results

### 3.1. Crack Distribution in Shear-Span and Failure Modes

With the increase of loads, shear cracks turned up successively at the shear-span, with clear splitting sounds due to the separation of expanded-shales intersected with the shear cracks and the pull-out of steel fibers from the matrix of the SFRELC. The shear failure of the test beams took place along a main shear crack, once formed. Accompanying the shear cracks, flexural cracks appeared firstly at the bottom of the pure bending segment and then extended slowly upward along the depth. As the test beams were designed with a larger ratio of longitudinal reinforcement to ensure the failure at the shear-span, the flexural cracks were secondary. When the maximum width of the shear cracks was up to 0.4 mm, the average and maximum width of flexural cracks was only 0.03–0.06 mm and 0.05–0.10 mm, respectively.

**Figure 2:**
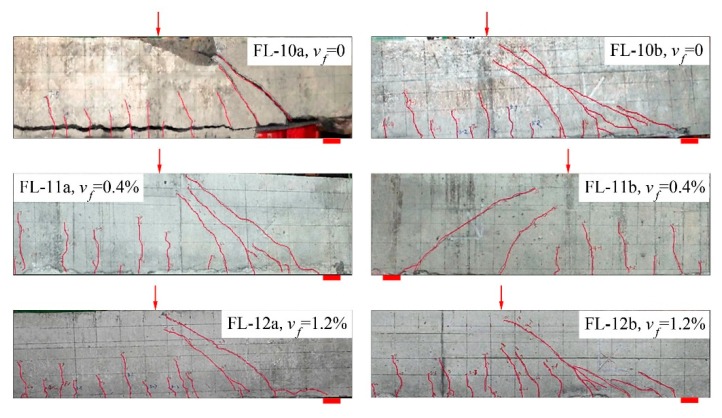
Crack distribution of test beams with different *v*_f_.

Figure 2 presents the crack distribution of test beams with a varying *v*_f_. With the same *λ* = 2.0, similar shear cracks appeared on the test beams, and the one near support developed to be the main shear crack, which resulted in the shear compression failure. The tearing crack on the FL-10a without steel fiber along the longitudinal rebars appeared abruptly; the bottom concrete cover came away and the shear failure formed quickly. With the increase of *v*_f_, the main shear crack kept away from the support with a more vertical distribution pattern, and more flexural-shear cracks appeared at the shear-span. This demonstrated that the shear cracking resistance and shear capacity in a region near support was strengthened due to the presence of steel fibers in SFRELC. As a result, the shear capacity of the test beams increased 25.1%, 35.9%, and 43.6% with *v*_f_ = 0.4%, 0.8%, and 1.2%, respectively, compared to that of test beams without steel fibers. By using the formulas of flexural capacity of reinforced SFRELC beams [17], the calculated ultimate moment of test beams with *v*_f_ = 1.2% is 132 kN·m, which corresponds to the shear force of 181 kN. Because the shear capacity of the test beams increased with the increase of *v*_f_, the inclined tensile failure of beam FL-12b with *v*_f_ = 1.2% tended to be accompanied by flexural characteristics. This is similar to the flexural failure achieved in the experimental studies of shear resistance for steel fiber reinforced-lightweight concrete beams and reinforced SFRC beams without web reinforcement [36,42]. Therefore, the failure of reinforced SFRELC beams without web reinforcement could be transferred from shear to flexure by using a higher volume fraction of steel fiber in SFRELC.

**Figure 3:**
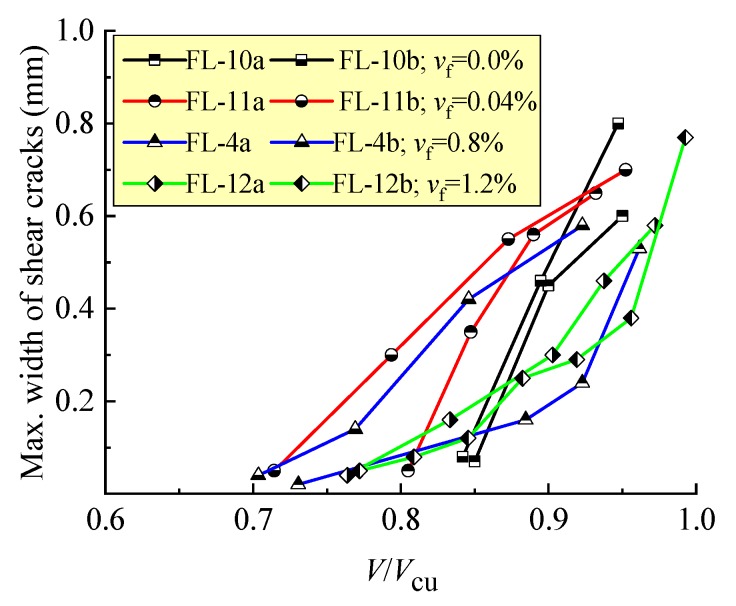
Maximum width of the shear crack on test beams with different *v*_f_.

As displayed in Figure 3, without steel fibers, the shear crack of the FL-10a and FL-10b beams initially appeared at a high loading level of *V*/*V*_cu_ about 85%, and rapidly extended to be a larger width than the control. When the steel fiber *v*_f_ = 0.4%, the status on FL-11a and FL-11b beams tended to be improved, but the discreteness of *V*/*V*_cu_ at the same maximum width of shear crack was higher in these two beams. With a further increase in *v*_f_, the confinement effect of the steel fibers on crack extension became more efficient, the shear crack appeared initially at a relative lower loading level *V*/*V*_cu_ below 77%, the main crack width opened continuously under the increasing load, and an obvious increment took place at a high loading level *V*/*V*_cu_. This result suggests a certain ductility of the shear failure of the beams.

**Figure 4:**
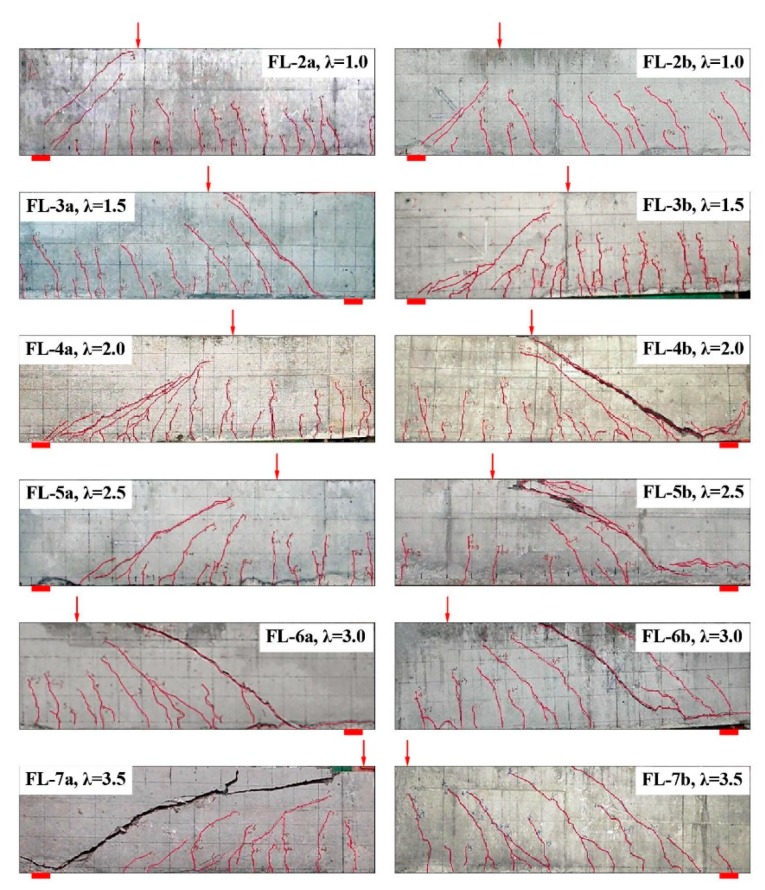
Crack distribution of test beams with varying *λ.*

Figure 4 presents the crack distribution of the test beams with a different shear-span to depth ratio *λ*. When *λ* = 1.0, two main inclined cracks appeared at the shear-span of the FL-2a and FL-2b beams with small widths during the loading process, and the inclined compression failure finally turned up with the characteristics of a crushed SFRELC at the top part of the inclined band formed between these two main inclined cracks. When 1.5 ≤ *λ* ≤ 2.5, the web shear cracks turned initially and extended to the supports and loading points along the inclined direction, and one of them developed as the main shear crack with the increasing load. The shear compression characteristics developed clearly with the crushed SFRELC in the shear compression zone accompanied by the shear cracks in the larger width within the tensile zone. When *λ* = 3.0 and 3.5, several shear cracks appeared almost parallel in the shear-span; one of them near the support quickly developed upward and downward to be the main inclined crack, and then the inclined tensile failure took place with the characteristics of two parts separated at the shear-span. If the load was exerted sequentially, the tearing crack of the SFRELC along the longitudinal rebars turned up on beams FL-6b and FL-7a with a loud sound. Generally, the shear crack distribution and failure mode of the test beams were mostly controlled by the shear-span to depth ratio, while the failure procedure was successive due to the confinement of the steel fibers.

**Figure 5:**
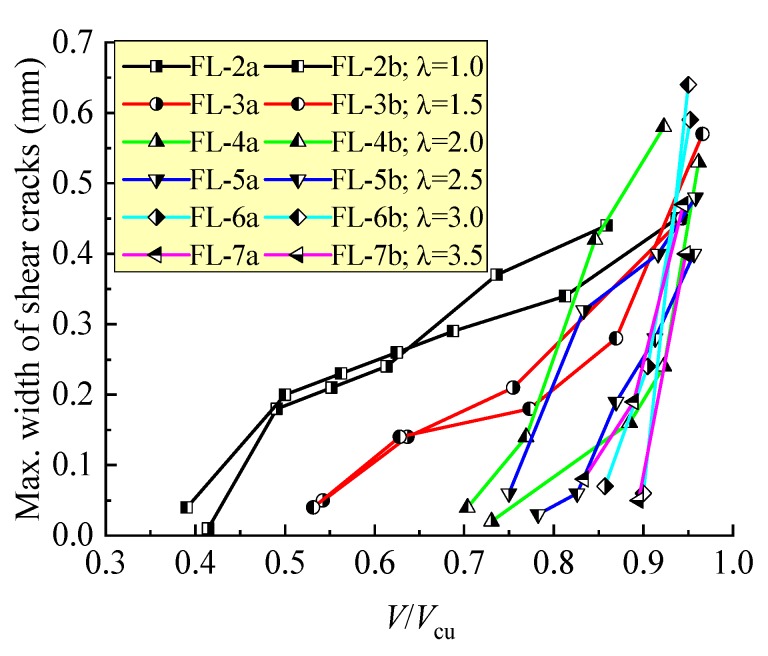
Maximum width of shear crack on test beams with a different *λ.*

In view of the test beams with varying *λ*, the changes of maximum width of shear cracks with the loading level *V*/*V*_cu_ are exhibited in Figure 5. This figure indicates the direct relationship between the shear crack opening and failure modes of the test beams. With the increase of *λ*, the loading level *V*/*V*_cu_ increased when the initial shear crack appeared, the extending of the main crack became fast under the increasing loads, and the failure tended to be abrupt. When *λ* ≤ 2.5, the process of the shear crack opening was persistent with the increase of the loading level *V*/*V*_cu_; an ominous sign of shear failure was clear due to the larger opening of the shear crack on the sides of the test beams. When *λ* ≥ 3, the initial shear crack turned up when the loading level *V*/*V*_cu_ was 83.3–90.0% and extended rapidly with a slightly increased load. This accompanied an abrupt inclined fracture of the beams.

### 3.2. Mid-Span Deflection

**Figure 6:**
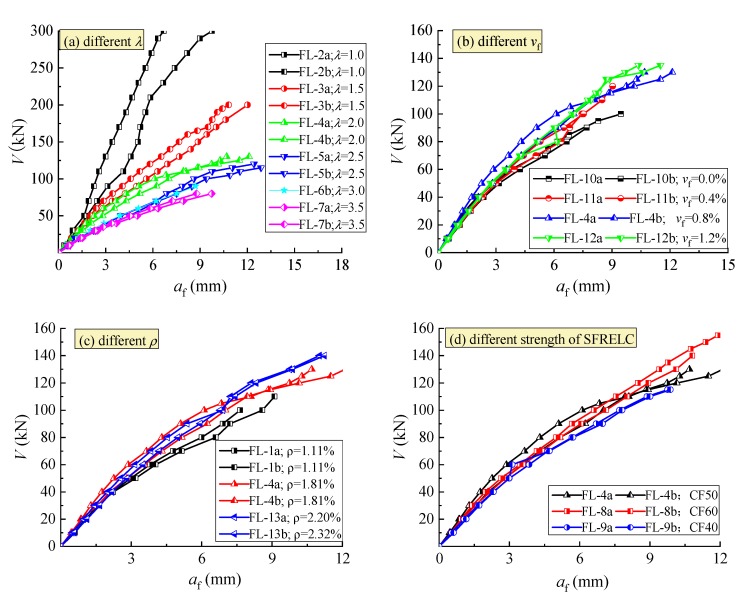
Load vs. mid-span deflection curve of test beams. (**a**) Different *λ*; (**b**) Different *v*_f_; (**c**) Different *ρ*; (**d**) Different strength of SFRELC.

The curves of shear force *V* with mid-span deflection *a*_f_ of the test beams are exhibited in Figure 6. The inflection point on the curves was not clearly recognizable. However, the different slope appeared with an increasing load. With the increase of *λ*, as presented in Figure 6a, the mid-span deflection increased under the same shear force due to the increased flexural deformation with the increased bending moment at the mid-span section, and the beam action became a controlling role in the shear resistance [31,38]. With the increase of *v*_f_, as presented in Figure 6b, the mid-span deflection decreased under the same shear force due to the increase of the flexural stiffness of the mid-span section and the structural entirety of the shear-span [12,13,14,17], and the beam action tended to be improved. With the increase of *ρ*, as presented in Figure 6c, the mid-span deflection reduced due to the increasing dowel action of longitudinal reinforcement, especially at a high loading level. The strength of SFRELC had little influence on the change of mid-span deflection at a lower loading level, but an obvious benefit appeared at a high loading level (Figure 6d). This result is due to the enhancement of steel fibers for SFRELC with a good bonding properties, which promoted the interlocking action of aggregates along the rough concrete surfaces on each side of the crack, and enhanced the dowel action of the longitudinal reinforcement [36,37,38].

## 4. Prediction of Shear Performances

### 4.1. Shear-Cracking Performance

To eliminate the influence of the sectional dimension, the shear stress *τ*_cr_ of the shear-cracking force on the unit sectional area was computed as,

(1) $$\tau_{\mathrm{cr}}=V_{\mathrm{cr}}/bh_{0}$$

**Figure 7:**
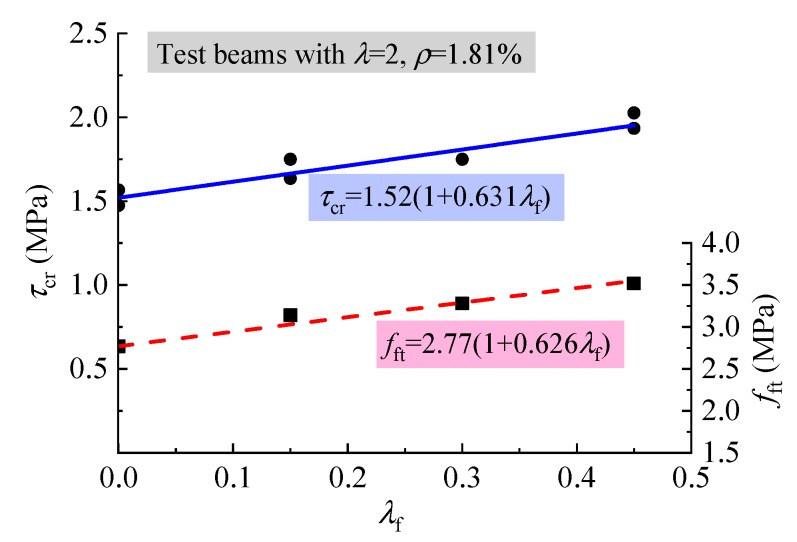
Variations of *τ*_cr_ and *f*_ft_ with *λ*_f_.

The fiber factor *λ*_f_ is usually used to represent the effect of steel fiber, which is the product of the aspect ratio *l*_f_/*d*_f_ with *v*_f_. Considering the respect ratio *l*_f_/*d*_f_ = 37.5 for the steel fiber used, the fiber factor *λ*_f_ = 37.5*v*_f_. The increase of *τ*_cr_ and *f*_ft_ with *λ*_f_ is exhibited in Figure 7. It can be seen from this figure that almost the same increment existed with the strengthening factor 0.631 and 0.626, respectively between *τ*_cr_ and *f*_ft_ with *λ*_f_, in which 1.52 and 2.77 are the initial values of the average *τ*_cr_ and *f*_ft_ of the test beams and SFRELC without steel fibers. This indicates that the strengthening of the shear cracking relies on the increase of tensile strength of SFRELC, and *τ*_cr_ varies in direct proportion to *f*_ft_. The same relationship was also obtained for reinforced SFRC beams without web reinforcement [39]. As the confinement effect of steel fibers on the micro-cracks in concrete improves the internal stress distribution of SFRELC at the shear span, and the concentrated stress at the micro-cracks is transferred farther by the successively distributed steel fibers [43], the shear cracking resistance of test beams can be promoted with the presence of steel fibers in SFRELC.

**Figure 8:**
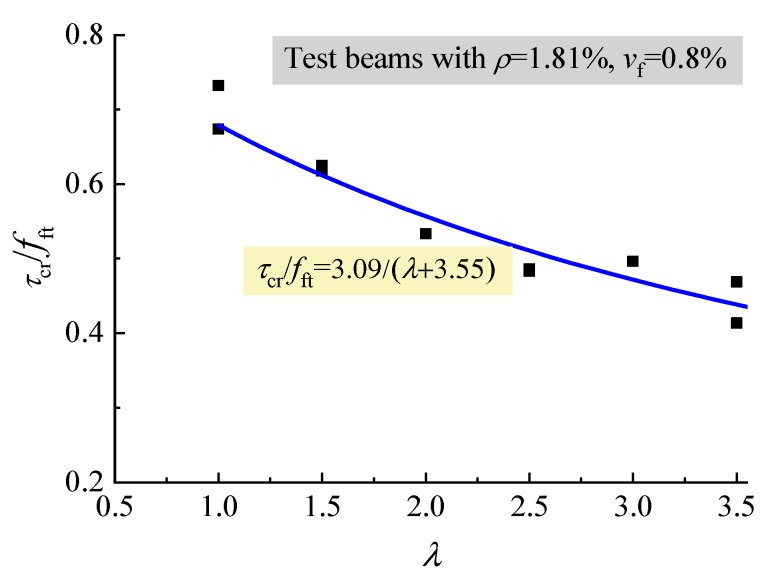
Variations of *τ*_cr_/*f*_ft_ with *λ*.

Figure 8 shows that the *τ*_cr_ decreased with the increase of *λ*. This result is due to the loading mechanism of reinforced concrete beams without web reinforcement no matter what kind of concrete is used [14,29,39].

Based on the analyses above, Equation (2), proposed by Zhao et al. [39] for the prediction of the shear-cracking resistance of reinforced SFRC beams without web reinforcement, can also be used for the prediction of the shear-cracking resistance of reinforced SFRELC beams without web reinforcement:

(2) $$\frac{V_{\mathrm{cr}}}{bh_{0}}=(\frac{2.45}{\lambda +3.5}+\frac{20\rho }{\lambda +1.1})f_{\mathrm{ft}}$$

where *λ* = 3.5 when *λ* ≥ 3.5, and *ρ* = 4.0% when *ρ* ≥ 4.0%.

**Figure 9:**
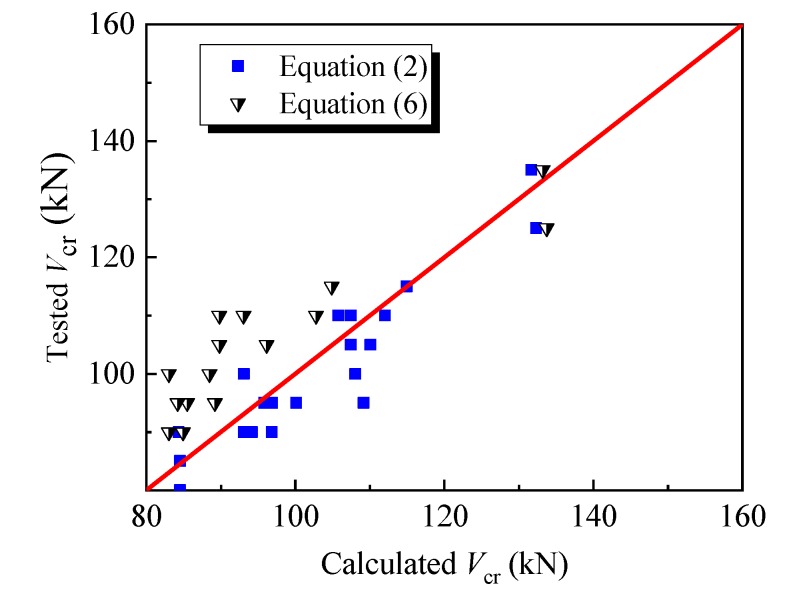
Comparison of tested and calculated values of *V*_cr_.

Figure 9 displays the comparison of tested *V*_cr_ to calculated *V*_cr_ with Equation (2). The mean value of tested to calculated values is 0.985 with a variation coefficient of 0.054.

Meanwhile, the test results were further fitted by the formula proposed by Rebeiz [29] for the calculation of the shear-cracking force of reinforced concrete beams without web reinforcement:

(3) $$\frac{V_{\mathrm{cr}}}{bh_{0}}=0.4+\sqrt{{f_{c}}^{\prime }\rho /\lambda }(2.7-0.4\alpha_{d})$$

where, *f*_c_′ is the cylindrical compressive strength of concrete. *α*_d_ is the adjustment factor, *α*_d_ = *λ* when 1.0 < *λ* < 2.5, and *α*_d_ = 2.5 when *λ* ≥ 2.5.

**Figure 10:**
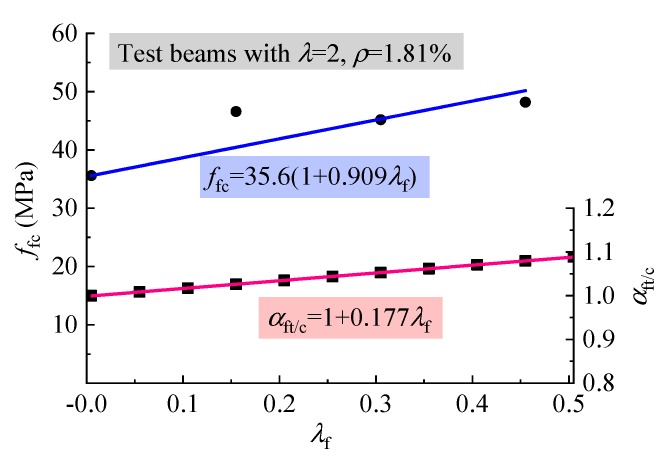
Variations of *f*_fc_ and *α*_ft/c_ with *λ*_f_.

The comparison results show that the mean ratio of tested *V*_cr_ to calculated *V*_cr_ with Equation (3) is 1.203 with a variation coefficient of 0.084, in which *f*_c_′ = 0.81 *f*_fc_ [34]. This shows that Equation (3) gives a lower predicted value of *V*_cr_ for reinforced SFRELC beams without web reinforcement. The reason for this result is mainly due to the different mechanisms of standard SFRELC specimens under compression and tension, which results in the insufficient representation of $\sqrt{{f_{c}}^{\prime }}$ to the strengthening effect of steel fiber on the tensile strength of SFRELC. Although *f*_fc_ of SFRELC increases obviously with *v*_f_ due to the effective confinement of steel fiber on the transversal deformation of SFRELC prismatic specimens, $\sqrt{{f_{c}}^{\prime }}$ could not catch up with the value of *f*_ft_ [5,6,7,42,44]. To allow Equation (3) to be used for predicting *V*_cr_ of reinforced SFRELC beams without web reinforcement, the difference between *f*_ft_ and *f*_fc_ strengthened by steel fibers should be considered. Therefore, the ratio of *f*_ft_ to $\sqrt{{f_{c}}^{\prime }}$ is calculated with the relationship represented in Figure 7 and Figure 10:

(4) $$\alpha_{\mathrm{ft}/c}=\left(1+0.626\lambda_{f}\right)/\sqrt{\left(1+0.909\lambda_{f}\right)}$$

Based on the numerical analysis presented in Figure 10, Equation (4) can be rewritten as

(5) $$\alpha_{\mathrm{ft}/c}=1+0.177\lambda_{f}$$

Then, Equation (3) can be revised as follows,

(6) $$\frac{V_{\mathrm{cr}}}{bh_{0}}=\alpha_{\mathrm{ft}/c}\left[0.4+\sqrt{{f_{c}}^{\prime }\rho /\lambda }(2.7-0.4\alpha_{d})\right]$$

The comparison results of tested *V*_cr_ to calculated *V*_cr_ with Equation (6) are also displayed in Figure 9. The mean value of tested to calculated values is 1.145 with a variation coefficient of 0.078. The lower predictive value of Equation (6) may be due to the different relationship between the *f*_c_′ and *f*_fc_ of SFRELC from that of conventional concrete. This should be further confirmed due to the lack of experimental data collected. Whatever the case, Equation (6) provides a way to modify the predictive formula of the shear-cracking resistance of reinforced concrete beams without web reinforcement to be used for that of the reinforced SFRELC beams. This can be done simply by linking that to the compressive and tensile strengths of SFRELC.

### 4.2. Shear Capacity

**Figure 11:**
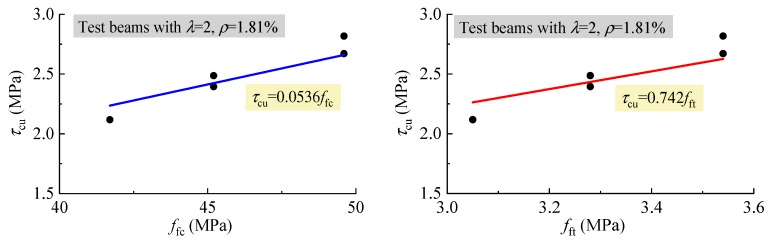
Variations of *τ*_cu_ with *f*_fc_ and *f*_ft._

**Figure 12:**
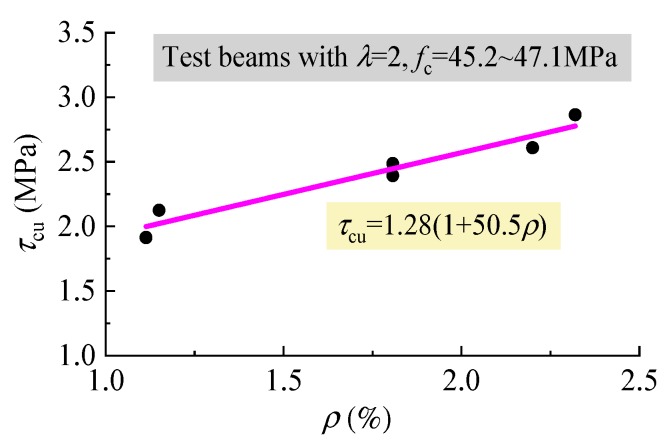
Variations of *τ*_cu_ with *ρ*.

In this study, under the similar conditions of other influencing factors, the average ultimate shear stress *τ*_cu_ = *V*_cu_/*bh*_0_ was in direct proportion to *f*_fc_ and *f*_ft_ as presented in Figure 11, and linearly increased with *ρ* as presented in Figure 12. This indicates that the similar regularities exist between *τ*_cu_ and *ρ*, *f*_fc_, *f*_ft_ for the reinforced SFRELC beams compared to the reinforced conventional and lightweight concrete beams, as in previous reports [18,22,27,28,34].

**Figure 13:**
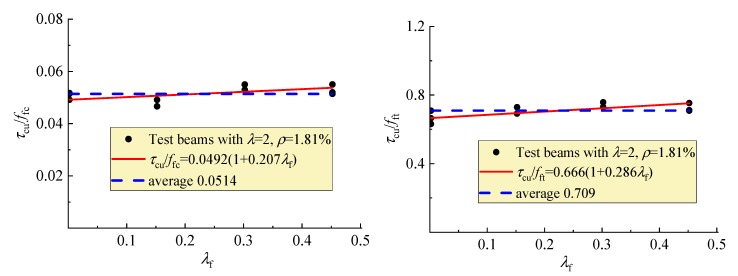
Variations of *τ*_cu_/*f*_fc_ and *τ*_cu_/*f*_ft_ with *λ*_f_.

Together, Figure 7, Figure 10, and Figure 13 shows that the strengthening effect of steel fiber on shear capacity is substantially implied in *f*_fc_ and *f*_ft_, although the *τ*_cu_/*f*_fc_ and *τ*_cu_/*f*_ft_ trend to increase with *λ*_f_. Therefore, the formulas for the calculation of shear capacity of reinforced lightweight-aggregate concrete, conventional concrete, or SFRC beams without web reinforcement are applied in this study.

Equation (7) was proposed by Li and Yu [27] and revised based on the experimental data of reinforced lightweight-aggregate concrete beams without web reinforcement [18,34],

(7) $$\frac{V_{\mathrm{cu}}}{bh_{0}}=\frac{0.024(2+100\rho )}{\lambda -0.3}f_{c}$$

In this formula, *λ* = 4 when *λ* ≥ 4, and *ρ* = 3.0% when *ρ* ≥ 3.0%. For the reinforced SFRELC beam, *f*_c_ is replaced by *f*_fc_.

Equation (8) was proposed by Li et al. [28] based on the experimental data of reinforced SFRC beams without web reinforcement:

(8) $$\frac{V_{\mathrm{cu}}}{bh_{0}}=\frac{0.115+0.192\lambda +28.7\rho }{\lambda -0.6}f_{\mathrm{ft}}$$

In this formula, *λ* = 4.5 when *λ* ≥ 4.5, and *ρ* = 4.0% when *ρ* ≥ 4.0%.

**Figure 14:**
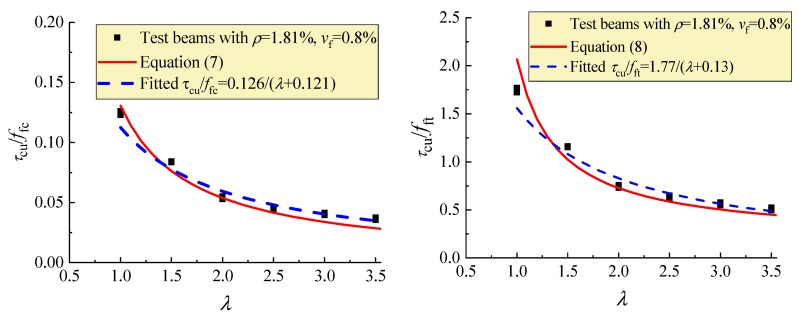
Variations of *τ*_cu_/*f*_c_ and *τ*_cu_/*f*_ft_ with *λ.*

Figure 14 presents the comparison of test data changed with *λ* to the fitted curves and the curves of Equations (7) and (8). This figure exhibits the good fitness of Equations (7) and (8) with the test data, and the consistent regularity between the fitted and calculated curves.

At the same time, Equation (9) proposed by Rebeiz [29] and Equation (10) proposed by Kim and Park [30] are also used to verify the test results of this study. For the convenience of description and comparison, the symbols with the same meaning are unified as much as possible.

(9) $$\frac{V_{\mathrm{cu}}}{bh_{0}}=0.4+\sqrt{{f_{c}}^{\prime }\rho /\lambda }(10-3\alpha_{d})$$

In this formula, *f*_c_′ and *α*_d_ are the same as in Equation (3):

(10) $$\frac{V_{\mathrm{cu}}}{bh_{0}}=3.5(\frac{1}{\sqrt{1+0.008h_{0}}}+0.18){f_{c}}^{\prime \alpha /3}\rho^{3/8}(0.4+\frac{1}{\lambda })$$

where *α* is a coefficient about failure mode. *a* = 2 − *λ*/3 when 1 ≤ *λ* < 3, and *α* = 1 when *λ* ≥ 3.

Taking *f*_c_′ = 0.81 *f*_fc_ of SFRELC into Equations (9) and (10), the calculated shear capacity can be computed.

With the shear capacity of each beam predicted by the formulas above, the ratio of the tested to predicted values can be computed. The statistical results for all beams of this study are listed in Table 2. The mean ratios are close to 1.0 with acceptable variation coefficients. This indicates that these formulas can be used to predict the shear capacity of reinforced SFRELC beams without web reinforcement. However, it should be noted that because Equations (8)–(10) are built for reinforced conventional concrete or SFRC beams, the ratios of FL-10a and FL-10b without steel fiber are 87.2%, 76.9% and 77.3% of the mean ratio, respectively. For the modified Equation (7), considering the difference between lightweight-aggregate concrete and conventional concrete, the ratio of FL-10a and FL-10b without steel fiber are 91.1% of the mean ratio. This demonstrates that the presence of steel fibers in SFRELC makes up for the shortcoming of expanded shales ruptured along cracks and enhances the interlocking action of expanded shales in the shear resistance.

## 5. Suggestion for Design of Shear Capacity

For the design of the shear capacity of reinforced concrete beams without web reinforcement, the standard worldwide formulas are all simplified to provide sufficient reliability of loading performance. In the standards CSA 23.3-04, Eurocode 2, and ACI 318-14, the effects of *λ* are not considered [19,20,21]. This results in a great difference between the tested and calculated shear capacities of the beams subjected to concentrated loads in this study. Therefore, the design formulas of reinforced lightweight-aggregate concrete beams specified in the China standard JGJ 12-2006 are used as the base [18], that is

(11) $$\frac{V_{\mathrm{cu}}}{bh_{0}}=\frac{1.5}{\lambda +1.0}\beta_{\rho }f_{t}$$

where, *β_ρ_* is the influencing coefficient of longitudinal reinforcement ratio, *β_ρ_* = (0.7 + 20 *ρ*). *f*_t_ is the tensile strength of the lightweight-aggregate concrete. Take *λ* = 1 when *λ* < 1, and *λ* = 4.0 when *λ* > 4.0.

Meanwhile, the formula proposed by Yi et al. [34] for the design of reinforced lightweight-aggregate concrete beams without web reinforcement is also selected to compare the test results, that is,

(12) $$\frac{V_{\mathrm{cu}}}{bh_{0}}=\frac{0.72}{\lambda -0.32}\beta_{\rho }f_{t}$$

where, *β_ρ_*, *f*_t_ and *λ* are the same as in Equation (11).

For the reinforced SFRELC beams, *f*_t_ in Equations (11) and (12) is replaced by *f*_ft_.

The formula proposed in ACI 544 [24] for the design of reinforced SFRC beams without web reinforcement is

(13) $$\frac{V_{\mathrm{cu}}}{bh_{0}}=\frac{2}{3\lambda^{0.25}}f_{\mathrm{ft}}$$

**Figure 15:**
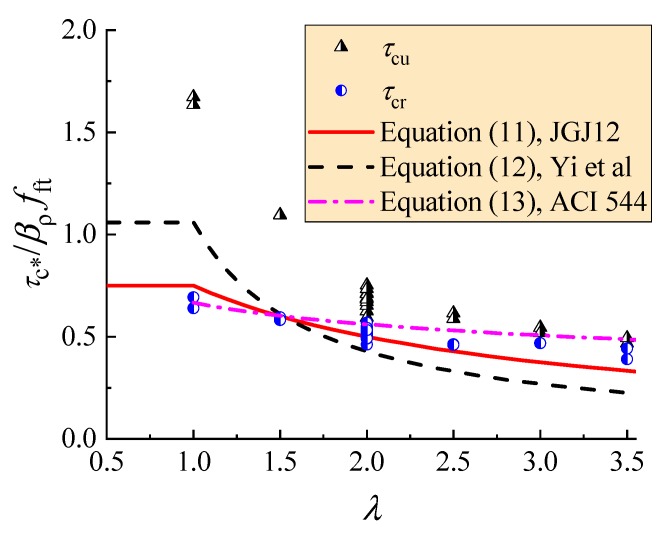
The comparison of predicted results to test data.

Figure 15 exhibits the comparison of Equations (11)–(13) to test data of the average ultimate shear stress *τ*_cu_ and the average shear-cracking stress *τ*_cr_. The equations are reliable to be used as a lower enveloped curve of tested *τ*_cu_ for the design of the shear capacity of reinforced SFRELC without web reinforcement. Comparatively, Equation (13) is much more conservative with its lowest calculated shear capacity than that of Equations (11) and (12) in the condition of *λ* < 1.5, while Equations (11) and (12) are much more conservative than Equation (13) in the condition of *λ* > 1.5.

Meanwhile, Equation (11) can also be used as a lower enveloped curve of tested *τ*_cr_ for the design of the shear cracking resistance of reinforced SFRELC without web reinforcement, while Equation (12) gives a shear force below the shear cracking resistance at *λ* > 1.5. That is, under the shear force designed by Equations (11) and (12), the reinforced SFRELC beams works without shear cracks.

## 6. Conclusions

Based on the experimental investigation, the conclusions can be drawn as follows:

(1) The presence of steel fiber in SFRELC promotes the shear performance of reinforced SFRELC beams without web reinforcement. With an increase of the volume fraction of steel fiber up to 1.2%, the dowel action was increased due to the elimination of the tearing crack along the longitudinal rebars; the main shear crack kept away from the support with a more vertical distribution pattern, and the beam action to shear resistance was improved with more flexural-shear cracks appearing at the shear-span segment. The loading level of the shear crack appeared initially reduced from about 85% to below 77% of the peak, the main crack width opened continuously under the increasing load, and the shear failure of the test beams took place with certain ductility. The shear capacity of the test beams increased 25.1%, 35.9%, and 43.6% with *v*_f_ = 0.4%, 0.8%, and 1.2%, respectively compared to that of the test beams without steel fibers. This suggests the possibility that the reinforced SFRELC beams without web reinforcement failed from shear to flexure by using a higher volume fraction of steel fiber.

(2) The distribution pattern of the shear cracks and the failure mode of reinforced SFRELC beams without web reinforcement were dominated by the shear-span to depth ratio. This could not be changed by the beneficial actions of steel fibers and longitudinal tensile reinforcement; despite this, the shear failure tended to be successive with lower brittleness. With the increase of *λ*, the beam action became a controlling role in the shear resistance.

(3) The strengthening of the shear-cracking resistance of the test beams relies on the increase of the tensile strength of SFRELC. That is, the shear-cracking resistance of the test beams is in direct proportion to the tensile strength of SFRELC. Based on the comparative analyses and the reasonable modifications to the formulas of SFRC and reinforced concrete beams, the predictive formulas for the shear-cracking resistance of reinforced SFRELC beams without web reinforcement are proposed.

(4) The presence of steel fibers in SFRELC makes up for the shortcoming of the expanded shales ruptured along cracks; the strengthening effect of steel fiber on the shear capacity of the test beams was substantially suggested by the compressive and tensile strengths of SFRELC. Replaced by the compressive and tensile strengths of SFRELC, the formulas for the shear capacity of reinforced lightweight-aggregate concrete beams, SFRC beams and conventional concrete beams without web reinforcement can be used to predict the shear capacity of reinforced SFRELC beams without web reinforcement. For the reliable design of reinforced SFRELC beams without web reinforcement, formulas for shear capacity represented as the lower enveloping curves of the test data are suggested.

## Figures and Tables

**Table 1:** Sectional dimension and influencing factor combination of test beams. SFRELC, steel fiber reinforced expanded-shale lightweight concrete.

Identifier	*b* (mm)	*h*_0_ (mm)	*a* (mm)	*ρ* (%)	*λ*	*v*_f_ (%)	Tested Strength of SFRELC (MPa)				Tested *V*_cr_ (kN)	Tested *V*_cu_ (kN)
							Grade	*f*_cu_	*f*_fc_	*f*_ft_		
FL-1a	155	364	728	1.11	2.0	0.8	CF50	58.2	47.1	3.32	95	108
FL-1b	150	364	728	1.15	2.0	0.8	CF50	58.2	47.1	3.32	90	116
FL-2a	152	362	362	1.78	1.0	0.8	CF50	58.6	47.0	3.35	135	326
FL-2b	153	362	362	1.77	1.0	0.8	CF50	58.6	47.0	3.35	125	320
FL-3a	155	362	543	1.75	1.5	0.8	CF50	54.8	45.2	3.28	115	212
FL-3b	150	362	543	1.81	1.5	0.8	CF50	54.8	45.2	3.28	110	207
FL-4a	150	362	724	1.81	2.0	0.8	CF50	54.8	45.2	3.28	95	130
FL-4b	150	362	724	1.81	2.0	0.8	CF50	54.8	45.2	3.28	95	135
FL-5a	154	362	905	1.76	2.5	0.8	CF50	58.2	47.1	3.32	90	120
FL-5b	155	362	905	1.75	2.5	0.8	CF50	58.2	47.1	3.32	90	115
FL-6a	150	362	1086	1.81	3.0	0.8	CF50	56.2	46.6	3.34	90	105
FL-6b	150	362	1086	1.81	3.0	0.8	CF50	56.2	46.6	3.34	90	100
FL-7a	150	362	1267	1.81	3.5	0.8	CF50	56.2	46.6	3.34	75	90
FL-7b	150	362	1267	1.81	3.5	0.8	CF50	56.2	46.6	3.34	85	95
FL-8a	152	362	724	1.78	2.0	0.8	CF60	60.0	49.6	3.54	95	155
FL-8b	150	362	724	1.81	2.0	0.8	CF60	60.0	49.6	3.54	100	145
FL-9a	150	362	724	1.81	2.0	0.8	CF40	48.0	41.7	3.05	90	115
FL-9b	150	362	724	1.81	2.0	0.8	CF40	48.0	41.7	3.05	100	115
FL-10a	150	362	724	1.81	2.0	0	CF50	42.0	35.6	2.77	85	100
FL-10b	150	362	724	1.81	2.0	0	CF50	42.0	35.6	2.77	80	95
FL-11a	150	362	724	1.81	2.0	0.4	CF50	56.3	46.6	3.14	95	118
FL-11b	152	362	724	1.78	2.0	0.4	CF50	56.3	46.6	3.14	90	126
FL-12a	150	362	724	1.81	2.0	1.2	CF50	59.2	48.2	3.52	110	144
FL-12b	150	362	724	1.81	2.0	1.2	CF50	59.2	48.2	3.52	105	136
FL-13a	155	361	722	2.20	2.0	0.8	CF50	58.6	47.0	3.35	105	146
FL-13b	147	361	722	2.32	2.0	0.8	CF50	58.6	47.0	3.35	110	152

**Table 2:** Comparison of the ratios of the tested to calculated shear capacity.

Identifier	*λ*	*v*_f_ (%)	Equation (7)	Equation (8)	Equation (9)	Equation (10)
FL-1a	2.0	0.8	0.925	0.986	0.853	0.949
FL-1b	2.0	0.8	1.014	1.080	0.934	1.040
FL-2a	1.0	0.8	0.972	0.864	0.961	1.055
FL-2b	1.0	0.8	0.951	0.846	0.940	1.032
FL-3a	1.5	0.8	1.115	1.146	0.946	1.111
FL-3b	1.5	0.8	1.107	1.135	0.941	1.107
FL-4a	2.0	0.8	0.985	1.004	0.887	1.006
FL-4b	2.0	0.8	1.023	1.043	0.921	1.045
FL-5a	2.5	0.8	1.1143	1.120	1.269	1.236
FL-5b	2.5	0.8	1.064	1.069	1.212	1.180
FL-6a	3.0	0.8	1.2264	1.148	1.215	1.473
FL-6b	3.0	0.8	1.168	1.094	1.157	1.403
FL-7a	3.5	0.8	1.246	1.102	1.102	1.351
FL-7b	3.5	0.8	1.315	1.163	1.163	1.426
FL-8a	2.0	0.8	1.063	1.102	1.008	1.142
FL-8b	2.0	0.8	1.002	1.038	0.950	1.077
FL-9a	2.0	0.8	0.945	0.955	0.812	0.923
FL-9b	2.0	0.8	0.945	0.955	0.812	0.923
FL-10a	2.0	0	0.962	0.915	0.754	0.861
FL-10b	2.0	0	0.914	0.869	0.717	0.818
FL-11a	2.0	0.4	0.867	0.952	0.794	0.901
FL-11b	2.0	0.4	0.920	1.010	0.842	0.954
FL-12a	2.0	1.2	1.024	1.036	0.955	1.083
FL-12b	2.0	1.2	0.967	0.979	0.902	1.023
FL-13a	2.0	0.8	0.936	0.965	0.873	1.001
FL-13b	2.0	0.8	0.999	1.028	0.937	1.077
Mean ratio	-	-	1.029	1.023	0.956	1.084
Variation coefficient	-	-	0.109	0.089	0.154	0.159
